# Mesenchymal stem cells of Systemic Sclerosis patients, derived from different sources, show a profibrotic microRNA profiling

**DOI:** 10.1038/s41598-019-43638-0

**Published:** 2019-05-09

**Authors:** Paola Di Benedetto, Noemi Panzera, Paola Cipriani, Valentina Mastroiaco, Alessandra Tessitore, Vasiliki Liakouli, Piero Ruscitti, Onorina Berardicurti, Francesco Carubbi, Giuliana Guggino, Andrea Bianchi, Antinisca Di Marco, Francesco Ciccia, Edoardo Alesse, Roberto Giacomelli

**Affiliations:** 1 Department of Biotechnological and Applied Clinical Sciences, Rheumatology Unit, School of Medicine, University of L’Aquila, L’Aquila, Italy; 2 Department of Biotechnological and Applied Clinical Sciences, University of L’Aquila, L’Aquila, Italy; 3 Department of Internal Medicine, Division of Rheumatology, University of Palermo, Palermo, Italy; 4 Department of Information Engineering, Computer Science and Mathematics, University of L’Aquila, L’Aquila, Italy

**Keywords:** Autoimmune diseases, Systemic sclerosis

## Abstract

Systemic Sclerosis (SSc) is a disease with limited therapeutic possibilities. Mesenchymal stem cells (MSCs)-therapy could be a promising therapeutic option, however the ideal MSCs source has not yet been found. To address this problem, we perform comparison between bone marrow (BM)-MSCs and adipose (A)-MSCs, by the miRs expression profile, to identify the gene modulation in these two MSCs source. MicroRNAs (miRs) are RNAs sequences, regulating gene expression and MSCs, derived from different tissues, may differently respond to the SSc microenvironment. The miRs array was used for the miRs profiling and by DIANA-mirPath tool we identified the biological functions of the dysregulated miRs. In SSc-BM-MSCs, 6 miRs were significantly down-regulated and 4 miRs up-regulated. In SSc-A-MSCs, 11 miRs were significantly down-regulated and 3 miRs up-regulated. Interestingly, in both the sources, the involved pathways included the senescence mechanisms and the pro-fibrotic behaviour. Furthermore, both the MSCs sources showed potential compensatory ability. A deeper knowledge of this miRs signature might give more information about some pathogenic steps of the disease and in the same time clarify the possible therapeutic role of autologous MSCs in the regenerative therapy in SSc.

## Introduction

SSc is a complex multisystem disorder, characterised by microvascular damage, dysregulation of innate and adaptive immunity, and generalized fibrosis in the skin and multiple organs^1–8^, such as lungs^9^, gastrointestinal tract^10^, kidneys^11^ and heart^12^. Due to the heterogeneity, in terms of extent, severity, and rate of progression, the optimal therapeutic interventions for SSc is still lacking and, to date, no disease-modifying agents are available^13^ and future options include regenerative therapies by using stem cells. Among these cells, MSCs are a subset of multipotent cells, which may be identified in a large variety of tissues, including bone marrow, placenta, umbilical cord, adipose tissue, teeth and menstrual fluid, largely involved in the reparative function after damage, thus suggesting their potential role in regenerative medicine^14–18^. Considering the pleiotropic effects of MSC, displaying immunomodulatory, angiogenic and antifibrotic capabilities, MSC-based therapy could counteract the three main pathogenic axes of SSc^17–19^. On these bases, MSCs isolated from SSc patients (SSc-MSCs) were largely studied and it is largely accepted that SSc-BM-MSCs strongly differentiate toward myofibroblasts, mainly after cross-talking with endothelial cells from SSc patients, thus confirming the link between the early vascular involvement of SSc and subsequent fibrosis^3^. Furthermore, SSc-BM-MSCs exhibit increased expression of senescent marker but maintaining immunosuppressive and immune-regulatory functions^20^. As far as SSc-A-MSCs are concerned, available literature did not show any alterations in both phenotype and differentiative potential^14^ between cells obtained from SSc patients and controls, although a decrease of proliferative activity as well as migration capacity have been reported^21^. On these bases, recent data suggest that, independent of the source, MSCs from SSc patients may be pre-committed in their differentiative behaviour toward myofibroblast, conditioned by both the cytokines and the metabolic milieu, specific of the disease^3,18,22,23^. These data partially mirror what showed in another autoimmune disease, the Systemic Lupus Erythematosus (SLE)^24^, and lead to speculate that in the field of regenerative medicine, an allogeneic rather than an autologous MSC-based therapy might be preferable for future treatments. Furthermore, it has been shown that allogenic MSCs infusion is a safe therapy for patients with autoimmune disease, including SSc patients^25^. In this developing setting, it is mandatory to better characterise the specific profile of MSCs derived from different sources, to understand if autologous cells may be used in SSc patients, for regenerative medicine. At present, the large majority of the molecular actors, controlling MSCs functions, are still largely unknown and recently a great importance has been recognised at a new class of non-coding RNAs, the so-called microRNAs (miRs), and the expression of characteristic miRs profiles has been implicated in the control of the normal development of specific system such as the cardiovascular tree, as well as in the pathogenesis of autoimmune diseases and cancer^26^. It is well known that, miRs down-regulation may result in a loss of their ability to inhibit target mRNA and, on the contrary, the miR upregulation may lead to an increase of target mRNA inhibition. MiRs control the gene expression by complementary base pairing with specific sites on the 3′-untranslated regions of their target gene mRNAs^27^. In the last years, specific miRs profiles, associated with different diseases, have been tested in *in silico* studies, to identify the panel of genes potentially controlled, and this tissue- and disease-specific miRs profiles often resulted more informative and discriminant than mRNA profiles^26^.

Thus, we used the miRs array approach to define the profile of MSCs derived from different sources in SSc patients, in order to assess the possible molecular differences between these cells. Furthermore, the possibility to predict different cells behaviours specifically related to the tissues used as source, was approached *in silico*. A better knowledge of phenotype and functions of these cells, associated with their stem plasticity, should be considered the first basic step to plan and develop any MSC-based therapy, and also to better understand the preliminary results obtained in the small case series of SSc patients treated with MSCs.

## Results

### Identification of miR expression profiling discriminating SSc-BM-MSCs and SSc-A-MSCs

The SSc-MSCs were isolated from SSc patients and the demographic and clinical characteristics of the patients are showed in Table 1.

**Table 1:** Clinical and demographic features of the 6 diffuse SSc patients.

Sex/Age (yrs)	Disease duration at skin biopsy (years from RP)	MRSS/score at skin biopsy	Autoantibodies	ILD	PAH	SCR	RP	DU
F/45	<1	08/2	ANA/Scl-70	No	No	No	Yes	No
F/35	<1	10/1	ANA/Scl-70	No	No	No	Yes	Yes
F/31	<1	07/2	ANA/Scl-70	No	No	No	Yes	No
F/40	<1	10/2	ANA/Scl-70	No	No	No	Yes	No
F/43	<1	06/1	ANA/Scl-70	No	No	No	Yes	No
F/34	<1	09/2	ANA/Scl-70	No	No	No	Yes	No

RP = Raynaud’s phenomenon; MRSS = modified Rodnan skin thickness score (maximum possible score 51); ILD: interstitial lung disease; ANA = antinuclear antibodies; Scl-70 = anti topoisomerase; PAH = pulmonary arterial hypertension. SCR = scleroderma renal crisis. DU = digital ulcers. The internal organs involvement is referred to the time of biopsies.

**Figure 1:**
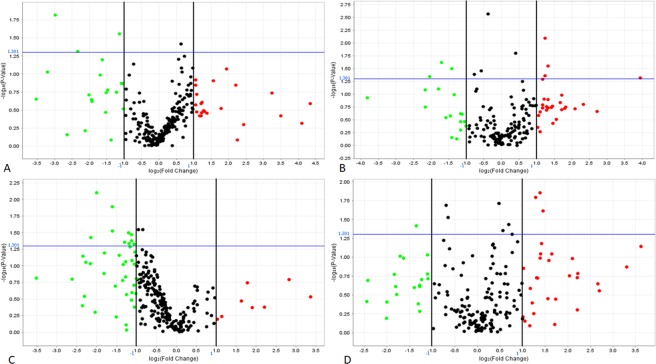
MiRs volcano plots. MiRs volcano plots of SSc-BM-MSC (**A,B**) and SSc-A-SSC (**C,D**). MicroRNAs’ distribution, obtained from TaqMan array human microRNA set A (in **A**,**C**) and set B (in **B**,**D**) was reported. In green are reported all the miRs down regulated, in red all the miRs up-regulated. Analyses were performed by using Expression Suite software. Y axis: p value (−log10), p = 0,05 threshold in blue. X axis: fold change (log2), black vertical lines indicate 0,5 (left) and 2 (right) fold change.

**Figure 2:**
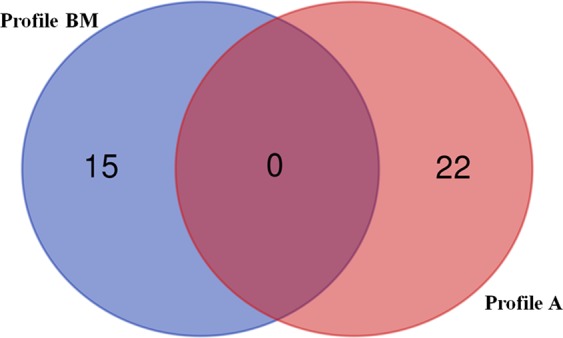
Venn diagram. Venn diagram shows the numbers of miRs expressed in Profile BM (blue) and the numbers of miRs expressed in Profile A (red). The Profile BM and Profile A do not share any miRs expression.

To compare miRs expression patterns among the MSCs derived from different source, we analysed the miRs expression levels by comparing SSc-BM-MSCs with HC-BM-MSCs, and SSc-A-MSCs with HC-A-MSCs as well. For each group, three replicates were analysed, and their expression levels are represented in Fig. 1. Among the 754 miRs analysed, we focused our analysis on those significantly dysregulated (p < 0,05), displaying more than 2-fold hypo- or hyper-expression (whole expression data are shown in Supplementary S1). SSc-BM-MSCs, showed 6 significantly down-regulated miRs, with fold change less than 0,5 (hsa-miR-200b-3p, hsa-miR-642a-5p, hsa-miR-483-3p, hsa-miR-519b-3p, hsa-miR-875-5p, hsa-miR-489-3p), 4 significantly up-regulated miRs, showing fold change higher than 2 (hsa-miR-10b-3p, hsa-miR-629-3p, hsa-miR-432-5p, hsa-miR-668-3p) and 5 miRs with less marked down- (fold change included between 0,584 and 0,767) or up- (fold change included between 1,324 and 1,55) regulation (hsa-miR-432-3p, hsa-miR-30d-3p, hsa-miR-335-3p, hsa-miR-30e-3p, hsa-miR-454-3p), when compared with HC-BM-MSCs. The SSc-A-MSCs showed 11 significantly down-regulated miRs, with fold change less than 0,5 (hsa-miR-218-5p, hsa-miR-135b-5p, hsa-miR-199b-5p, hsa-miR-204-5p, dme-mir-7, hsa-miR-500a-5p, hsa-miR-423-5p, hsa-miR-146a-5p, hsa-miR-708-5p, hsa-miR-155-5p, hsa-miR-146b-5p), 3 significantly up-regulated miRs, with fold change higher than 2 (hsa-miR-1227-3p, hsa-miR-1225-3p, hsa-miR-1267) and 8 miRs (hsa-miR-185-5p, hsa-miR-10a-5p, hsa-miR-222-5p, hsa-miR-766-3p, hsa-miR-1274A, hsa-miR-1271-5p, hsa-miR-27a-5p, hsa-miR-573) whose down- or up-regulation was less marked and included between 0,518 and 0,64 or 1,391 and 1,703, when compared with HC-A-MSCs (Table 2). Those results highlight 2 different miR profiles: Profile BM and Profile A, and the Venn diagram (Fig. 2) shows that the Profile BM and Profile A are characterized by totally different miRs expression.

**Table 2:** Dysregulated miRs in Profile BM and A.

Profile BM: SSc-BM-MSCs vs HC-BM-MSCs	RQ: relative quantification	p
miRs (miRbase ID)		
hsa-miR-200b-3p	0,126	0,015
hsa-miR-642a-5p	0,198	0,048
hsa-miR-483-3p	0,242	0,045
hsa-miR-519b-3p	0,307	0,024
hsa-miR-875-5p	0,377	0,032
hsa-miR-489-3p	0,456	0,028
hsa-miR-432-3p	0,584	0,041
hsa-miR-30d-3p	0,673	0,035
hsa-miR-335-3p	0,767	0,003
hsa-miR-30e-3p	1,324	0,016
hsa-miR-454-3p	1,550	0,038
hsa-miR-10b-3p	2,351	0,044
hsa-miR-629-3p	2,361	0,008
hsa-miR-432-5p	2,500	0,028
hsa-miR-668-3p	15,389	0,048
**Profile A: SSc-A-MSCs vs HC-A-MSCs**	**RQ: relative quantification**	**p**
**miRs (miRbase ID)**		
hsa-miR-218-5p	0,226	0,037
hsa-miR-135b-5p	0,250	0,008
hsa-miR-199b-5p	0,328	0,013
hsa-miR-204-5p	0,329	0,03
dme-mir-7	0,393	0,038
hsa-miR-500a-5p	0,406	0,044
hsa-miR-423-5p	0,436	0,032
hsa-miR-146a-5p	0,439	0,046
hsa-miR-708-5p	0,458	0,042
hsa-miR-155-5p	0,459	0,034
hsa-miR-146b-5p	0,480	0,047
hsa-miR-185-5p	0,518	0,029
hsa-miR-10a-5p	0,557	0,029
hsa-miR-222-5p	0,620	0,02
hsa-miR-766-3p	0,640	0,03
hsa-miR-1274A	1,391	0,019
hsa-miR-1271-5p	1,480	0,044
hsa-miR-27a-5p	1,621	0,037
hsa-miR-573	1,703	0,049
hsa-miR-1227-3p	2,445	0,016
hsa-miR-1225-3p	2,608	0,014
hsa-miR-1267	2,742	0,024

List of significantly dysregulated miRs in Profile BM and A. Significant miRs expression changes were identified using a threshold of p < 0,05 (p value calculation based on 2^−dCt^).

### MiRs target prediction analysis

*In silico* analysis, predicting the putative biological functions of the miRs, suggested their involvement in specific Kyoto Encyclopedia of Genes and Genomes (KEGG) signalling pathways. The KEGG database identifies significantly enriched metabolic pathways or signal transduction pathways from lists of candidate target genes and compares this enrichment with a reference background.

In the Profile BM, the 6 down-regulated miRs were implicated in 32 KEGG biological pathways (p value threshold 0,05). Among them, we focused the analysis on: Signalling Pathways Regulating Pluripotency Of Stem Cells (hsa04550, p = 0,009), MAPK Signalling Pathway (hsa04010, p = 0,01), HIF-1 Signalling Pathway (hsa04066, p = 0,01), TGF-beta Signalling Pathway (hsa04350, p = 0,02), p53 Signalling Pathway (hsa04115, p = 0,03), PI3K-Akt Signalling Pathway (hsa04151, p = 0,04). The 4 miRs up-regulated in Profile BM were involved in 1 KEGG pathway (p value threshold 0,05), the ECM-Receptor Interaction (hsa04512; p = 2,1 × 10^−11^).

In the Profile A, the 11 down-regulated miRs were identified in 18 KEGG pathways (p-value threshold 0,05), including: TGF-beta Signalling Pathway (hsa04350, p = 2 × 10^−5^), Signalling Pathways Regulating Pluripotency Of Stem Cells (hsa04550, p = 0,001), Regulation Of Actin Cytoskeleton (hsa04810, p = 0,008) and Wnt Signalling Pathway (hsa04310, p = 0,03). Furthermore, the 3 up-regulated miRs were involved in 1 KEGG biological process, the Thyroid Hormone Signalling Pathway (hsa04919, p = 0,007). Each pathway was reported in Table 3.

**Table 3:** KEGG pathways.

Profile BM	6 miR down-regulated		KEGG pathways	p-value	genes	miRs
		1.	Proteoglycans In Cancer (Hsa05205)	6,9e-05	40	5
		2.	Glioma (Hsa05214)	6,9e-05	18	5
		3.	Gap Junction (Hsa04540)	6,9e-05	22	6
		4.	Biotin Metabolism (Hsa00780)	0,0002	1	1
		5.	Erbb Signalling Pathway (Hsa04012)	0,0003	24	5
		6.	Renal Cell Carcinoma (Hsa05211)	0,0004	19	5
		7.	Neurotrophin Signalling Pathway (Hsa04722)	0,0005	31	5
		8.	Hepatitis B (Hsa05161)	0,002	30	6
		9.	Prostate Cancer (Hsa05215)	0,002	24	6
		10.	Phosphatidylinositol Signalling System (Hsa04070)	0,002	16	5
		11.	Glycosaminoglycan Biosynthesis - Chondroitin Sulfate / Dermatan Sulfate (Hsa00532)	0,004	3	2
		12.	Inositol Phosphate Metabolism (Hsa00562)	0,007	13	4
		13.	Lysine Degradation (Hsa00310)	0,007	9	4
		14.	Glycosphingolipid Biosynthesis - Lacto And Neolacto Series (Hsa00601)	0,009	5	2
		15.	Axon Guidance (Hsa04360)	0,009	26	3
		16.	Signalling Pathways Regulating Pluripotency Of Stem Cells (Hsa04550)	0,009	28	5
		17.	Chronic Myeloid Leukemia (Hsa05220)	0,01	19	5
		18.	Estrogen Signalling Pathway (Hsa04915)	0,01	19	6
		19.	Foxo Signalling Pathway (Hsa04068)	0,01	30	5
		20.	Thyroid Hormone Signalling Pathway (Hsa04919)	0,01	21	5
		21.	Pancreatic Cancer (Hsa05212)	0,01	17	5
		22.	MAPK Signalling Pathway (Hsa04010)	0,01	47	5
		23.	RNA Degradation (Hsa03018)	0,01	20	6
		24.	Acute Myeloid Leukemia (Hsa05221)	0,01	15	5
		25.	HIF-1 Signalling Pathway (Hsa04066)	0,01	23	6
		26.	Oocyte Meiosis (Hsa04114)	0,02	22	5
		27.	Endocytosis (Hsa04144)	0,02	37	5
		28.	TGF-Beta Signalling Pathway (Hsa04350)	0,02	15	4
		29.	Pathways In Cancer (Hsa05200)	0,02	66	6
		30.	P53 Signalling Pathway (Hsa04115)	0,03	16	4
		31.	PI3K-Akt Signalling Pathway (Hsa04151)	0,04	55	6
		32.	Melanoma (Hsa05218)	0,04	17	5
**Profile BM**	**4 miR up-regulated**		**KEGG pathway**	**p-value**	**genes**	**miRs**
		1.	ECM-Receptor Interaction (Hsa04512)	2,1 e-11	10	2
**Profile A**	**11 miR down-regulated**		**KEGG pathways**	**p-value**	**genes**	**miRs**
		1.	TGF-Beta Signalling Pathway (Hsa04350)	2,0E-05	21	8
		2.	Foxo Signalling Pathway (Hsa04068)	1,1E-03	38	9
		3.	Signalling Pathways Regulating Pluripotency Of Stem Cells (Hsa04550)	1,1E-03	36	10
		4.	Regulation Of Actin Cytoskeleton (Hsa04810)	7,9E-03	55	10
		5.	Thyroid Hormone Signalling Pathway (Hsa04919)	7,9E-03	26	10
		6.	Amphetamine Addiction (Hsa05031)	1,0E-02	18	9
		7.	Estrogen Signalling Pathway (Hsa04915)	1,0E-02	27	10
		8.	Choline Metabolism In Cancer (Hsa05231)	1,2E-02	29	10
		9.	Erbb Signalling Pathway (Hsa04012)	1,4E-02	25	9
		10.	Ras Signalling Pathway (Hsa04014)	1,6E-02	48	10
		11.	Thyroid Hormone Synthesis (Hsa04918)	1,9E-02	14	9
		12.	Ubiquitin Mediated Proteolysis (Hsa04120)	2,3E-02	32	10
		13.	Proteoglycans In Cancer (Hsa05205)	2,3E-02	44	10
		14.	Wnt Signalling Pathway (Hsa04310)	3,3E-02	30	10
		15.	Axon Guidance (Hsa04360)	3,5E-02	29	10
		16.	Prolactin Signalling Pathway (Hsa04917)	3,7E-02	19	8
		17.	Insulin Signalling Pathway (Hsa04910)	3,7E-02	35	10
		18.	Neurotrophin Signalling Pathway (Hsa04722)	4,7E-02	29	9
**Profile A**	**3 miR up-regulated**		**KEGG pathway**	**p-value**	**genes**	**miRs**
		1.	Thyroid Hormone Signalling Pathway (Hsa04919)	6,9E-03	6	2

KEGG pathways involving the dysregulated miRs. The p-value was calculated by DIANA miRPath v.3 software.

### Experimental validated target prediction

Using microT-CDS, we reported in Supplementary S2, all the predicted gene targeting the miRs including in the selected pathways. Furthermore, we restricted our investigation only on target genes, experimentally validated. None validated target gene has been found for miR up-regulated in both Profile BM and A. As far as the miRs down regulated in the Profile BM are concerned, the target genes experimentally validated were reported in Table 4. MiRs-200b-3b targets the validated genes: SMAD2/5, KLF4, CRKL, RASA2, ELK4, PAK2, TAOK1, JUN, RPS6KA3, RAP1B, DUSP1, PLCG1, FLT1, EIF4E2, PPP2R1B, ZMAT3, CDK2, PMAIP1, CCNE2, SESN1, SIAH1, PPP2R5E, YWHAG, BRCA1, PTK2, ITGAV, EFNA1, KDR, FLT1; miR-519a-3p targets HSPA8 and PTEN and the miR-483-3p targets ZAK. In the Profile A, the down regulated miRs are involved as follow: miR-155-5p targets SP1, JARID2, GSK3B, KAT6A, KRAS, PAK2/7, FGF9, CHD8 and CSNK1A1; miR-500a-5p targets RPS6KB1 and TBL1XR1; miR-146a-5p targets RBL1 and SMAD4; miR-146b-5p interacts with RBL1 and ARPC5; the miR-204-5p with FZD1, MEIS1 and CCND2; miR-423-5p targets ARPC5, ITGA5 and PRKACA; miR-135b-5p: SMARCAD1 and WASF2; miR-218-5p targets PPP1CC, ACTN1 and SENP2; the hsa-miR-199b-5p targets GIT1; and finally, the miR-708-5p targets: IQGAP1 and DIAPH1.

**Table 4:** Gene interactions experimentally validated.

Profile BM (miR down-regulated):	hsa-miR-200b-3p	hsa-miR-519a-3p,	hsa-miR-483-3p							
Signalling Pathways Regulating Pluripotency Of Stem Cells (Hsa04550)	SMAD2/5									
	KLF4									
MAPK Signalling Pathway (Hsa04010)	CRKL	HSPA8	ZAK							
	RASA2									
	ELK4									
	PAK2									
	TAOK1									
	JUN									
	RPS6KA3									
	RAP1B									
	DUSP1									
HIF-1 Signalling Pathway (Hsa04066)	PLCG1									
	FLT1									
	EIF4E2									
TGF-Beta Signalling Pathway (Hsa04350)	SMAD2/5									
	PPP2R1B									
P53 Signalling Pathway (Hsa04115)	ZMAT3	PTEN								
	CDK2									
	PMAIP1									
	CCNE2									
	SESN1									
	SIAH1									
PI3K-Akt Signalling Pathway (Hsa04151)	PPP2R5E	PTEN								
	YWHAG									
	CCNE2									
	CDK2									
	BRCA1									
	EIF4E2									
	PTK2									
	ITGAV									
	PPP2R1B									
	EFNA1									
	KDR									
	FLT1									
**Profile A (miRs down-regulated):**	**hsa-miR-155-5p**	**hsa-miR-500a-5p**	**hsa-miR-146a-5p**	**hsa-miR-146b-5p**	**hsa-miR-204-5p**	**hsa-miR-423-5p**	**hsa-miR-135b-5p**	**hsa-miR-218-5p**	**hsa-miR-199b-5p**	**hsa-miR-708-5p**
TGF-Beta Signalling Pathway (Hsa04350)	SP1	RPS6KB1	RBL1	RBL1						
			SMAD4							
Signalling Pathways Regulating Pluripotency Of Stem Cells (Hsa04550)	JARID2		SMAD4		FZD1		SMARCAD1			
	GSK3B				MEIS1					
	KAT6A									
	KRAS									
Regulation Of Actin Cytoskeleton (Hsa04810)	PAK2			ARPC5		ARPC5	WASF2	PPP1CC	GIT1	IQGAP1
	PAK7					ITGA5		ACTN1		DIAPH1
	KRAS									
	FGF9									
Wnt Signalling Pathway (Hsa04310)	GSK3B	TBL1XR1	SMAD4		CCND2	PRKACA		SENP2		
	CHD8				FZD1					
	CSNK1A1									

The genes target experimentally validated of the down-regulated miRs.

**Figure 3:**
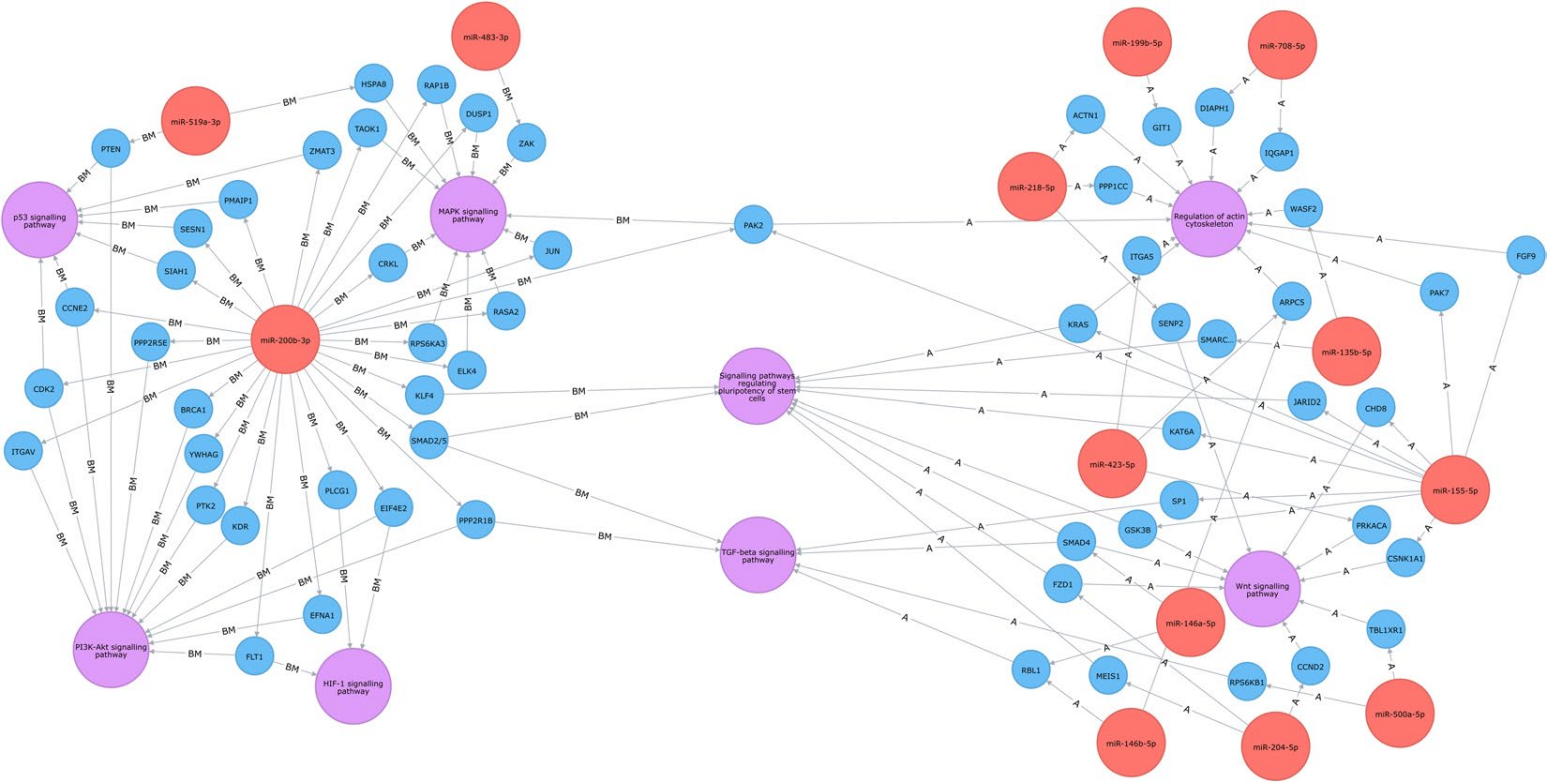
Graphical visualization of miRNA-target interactions. Neo4J software graphical visualization of miRNA-target interactions, concerning the Profile BM (left) and Profile (right) A. Red circles represent miRNAs. Blue circles represent genes that interact with the miRs, experimentally supported in TarBase. Purple circles represent the relevant pathways, that include validated target genes. Both the Profile BM and Profile A share “TGF-beta Signalling Pathway” and “Signaling Pathways Regulating Pluripotency Of Stem Cells”.

The results obtained were included in a Neo4J graph database^28^ to provide a graphical representation of miRNAs-targets interactions (Fig. 3). This graphical representation highlighted that both the Profiles shared, 2 important pathways in this clinical setting: the TGF-beta Signalling Pathway and Signalling Pathways Regulating Pluripotency Of Stem Cells.

## Discussion

Considering the regenerative potential of MSC, displaying immunomodulatory, angiogenic and antifibrotic capabilities, MSC-based therapy could counteract the three main pathogenic axes of SSc^17,22,29,30^, a clinical setting lacking effective therapy. This is the first paper reporting *in silico* comparative analysis of miRs profile of MSCs isolated from different sources (BM and A) of SSc patients. We showed that, independent from the source, an upregulation of the pathways regulating the senescence and the profibrotic phenotype, may be observed in the MSCs of patients affected by SSc, a disease characterized by diffuse fibrosis of skin and internal organs. Furthermore, both BM- and A-SSc-MSCs display a down regulation of the miRs controlling the genes related to cells survival, thus suggesting the ability of these cells to protect themselves, activating specific pathways in response to the critical conditions, found in the scleroderma microenvironment^31,32^, such as hypoxia and inflammation^14,33^. At present, one third of the clinical trials using engrafting MSCs are designed to evaluate their therapeutic role in autoimmune diseases (for the latest update, see ttp://www.clinicaltrials.gov). Considering the immunomodulatory, pro-angiogenic and antifibrotic capabilities of MSCs, their transplant may have a potential application in cell-based therapies for SSc and results obtained in preclinical models^3,6,14,19,20,23^, as well as the few cases reported in the SSc^34,35^ seem very promising. However, many *in vitro* experiments showed some differences in the biologic functions of SSc-MSCs, deriving from different tissues^3,6,22,23^. These data raised some concern about their efficacy in transplant strategy, since the profibrotic signature of SSc-MSCs may thwart their potential beneficial effects and suggested further refinements in the molecular and genetic knowledge of MSCs.

It has been reported that long‐term culture evokes continuous changes in MSCs: proliferation rate decays, the cell size increases, differentiation potential is affected, chromosomal instabilities may arise, and molecular changes are acquired. In fact, early passage MSCs were preferred for therapeutic efficacy in many clinical trials^36^ and, on this basis, we choose to characterize the miR profiling of P3 MSCs, providing a profiling of *in vitro* expanded MSCs at the time in which they may be used for transplantation. We observed that P3 SSc-BM-MSCs expressed 6 miRs significantly down-regulated and 4 miRs significantly up-regulated when compared with HC-BM-MSCs. As far as, A-tissue is concerned, P3 SSc-MSCs expressed 11 down-regulated miRs and 3 up-regulated, when compared with HC. These data identified 2 different profiles and of note, the miRs listed in one Profile were not present in the other Profile. This datum confirms that miRs constitute a family of regulators for gene expression, which may be largely tissue-dependent^37^.

To assess the biological significance of miRs listed in both Profiles, we relied on *in silico* analysis for predicting potential miRs targets. In both Profiles, we observed two specific patterns and by the “Diana miRPath” software, we try to identify the signalling pathways, significantly associated with each pattern in both Profiles.

In the Profile BM, 6 miRs were down-regulated, targeting 32 KEGG pathways, considering the high number of these pathways, we focused our discussion on the KEGG pathways that may play a specific relevance in SSc, such as “Signalling Pathways Regulating Pluripotency Of Stem Cells”, “MAPK Signalling Pathway”, “HIF-1 Signalling Pathway”, “TGF-Beta Signalling Pathway”, “P53 Signalling Pathway” And “PI3K-Akt Signalling Pathway”. The “Signalling Pathways Regulating Pluripotency Of Stem Cells” may involve transcription factors and their downstream target genes promoting self-renewal and pluripotency of stem cells. Using microT-CDS, among the validated target genes of this pathway, we observed the genes regulating for: SMAD-2 and -5, thus suggesting a profibrotic signature of MSCs^5^ and KLF4^38,39^, which is involved in the cellular senescence. The “MAPK Signalling Pathway” is involved in various cellular functions, including cell response to different stimulation. This pathway includes several validated target genes: HSPA8, TAOK1, RPS6KA3 and PAK2, regulating the adaptation to different stress stimuli and cells survival^29,40–42^, and CRKL, promoting activities including cytoskeletal remodelling, cell motility, cell proliferation and mitosis^38^. Furthermore, several genes involved in this pathway display pro-apoptotic activity, such as ELK4, JUN, RASA2, DUSP1 and ZAK, and proliferation, such as RAP1B^38^.

The “HIF-1 Signalling Pathway” includes gene encoding proteins, mediating adaptive responses to nitric oxide and reduced oxygen availability, a common condition during SSc, in which a desertification of vascular tree is present, and it is not surprising to observe that the validated target genes for this pathway are: PLCG1, FLT1 and EIF4E2, which may mediate MSCs proliferation, angiogenesis and survival in hypoxia condition^38,43,44^, suggesting a compensatory mechanism in these patients. The “TGF-beta Signalling Pathway” includes genes with a specific relevance during fibrotic process. The experimentally validated target genes are: SMAD2 and 5 and PPP2R1B, promoting the TGF-beta signalling^5,45^. The “p53 Signalling Pathway” may regulate genes involved in DNA damage and oxidative stress response. The experimentally validated target genes included in this pathway were: CDK2, CCNE2^46^ and SESN1, stimulating proliferation, and ZMAT3, PMAIP1, SIAH1, and PTEN^38^, contributing to apoptosis or cell-cycle inhibitor. The “PI3K-Akt Signalling Pathway” is activated by many cellular stimuli or toxic insults and regulates fundamental cellular functions such as transcription, translation, proliferation, growth, and survival. The validated target genes are: BRCA1 and EIF4E2, promoting cells response to damage and hypoxia^32,44^; YWHAG, promoting a more mature phenotype in MSCs^47^, CDK2 and CCNE2, playing a role in cell cycle G1/S transition^46^; PTK2, promoting proliferation; ITGAV and PPP2R1B, promoting the TGF-beta signalling in both normoxia and hypoxia; PTEN, PPP2R5E, regulating the balance between survival and apoptosis; EFNA1, KDR and FLT1, encoding member of the vascular endothelial growth factor family^38^.

In the Profile A, we listed 11 down-regulated miRs, targeting 18 KEGG pathways. Interestingly, 2 relevant target pathways, the “TGF-beta Signalling Pathway” and “Signalling Pathways Regulating Pluripotency Of Stem Cells” are the same observed in the Profile BM, although these pathways, in Profile A, are regulated by different miRs. The validated target genes, included in “TGF-beta Signalling Pathway” in Profile A are: SP1, SMAD4 and RPS6KB1, which may promote a more mature and profibrotic phenotype in MSCs^48,49^ and RBL1, which may inhibit the cells proliferation by arresting the cells in G1^50^. The validated target genes, included in “Signalling Pathways Regulating Pluripotency Of Stem Cells” are: JARID2, promoting more mature phenotype in MSCs; SMAD4, pro-fibrotic^3,51^; KAT6A and GSK3B, promoting cell proliferation^52,53^; MEIS1 and FZD1, promoting senescence^54,55^ and KRAS, promoting cell survival^56^ and SMARCAD1, which may play a role during DNA repair^38^. Some down-regulated miRs, in Profile A, target specific pathways, which have not been identified in Profile BM. “The Regulation Of Actin Cytoskeleton”, involving the validated target gene: ARPC5, ITGA5, WASF2, ACTN1, GIT1, IQGAP1 and DIAPH1 promoting actin cytoskeleton reorganization for cell invasion; PAK2/7, FGF9, PPP1CC and KRAS, promoting proliferation^38^, migration, and survival^42,56^, and of note, the target gene PAK2, promoting the cellular survival, was shared between BM- and A-MSCs. The “Wnt Signalling Pathway” including the validated genes: CHD8, GSK3B, TBL1XR1 and CCND2, promoting proliferation, migration and invasion; FZD1, promoting senescence; CSNK1A1, promoting self-renewal; and SMAD4 promoting a more mature phenotype in MSCs and SENP2, that may contribute to escape to TGF-beta signalling and PRKACA a survival kinase^38^.

Recently, it has been reported that perivascular cells, overexpressing the isoform 12 of A Disintegrin And Metalloprotease (ADAM12), play a role during fibrotic stimuli, trans-differentiating toward activated myofibroblast. Genetic ablation of ADAM12+ cells may limit the generation of profibrotic cells^57^. In previous work, we provide evidence of activated ADAM12 expression, in BM-SSc-MSC, suggesting their commitment toward a profibrotic activity^16^. Interestingly, we found that ADAM12 may be putatively targeted by several miRs (miR-200b-3p, -642a-5p, 483-3p, 519b-3p, -10b-3p, -629-3p, -432-5p, -668-ep in the BM profile; miR-135b-5p, -204-5p, -155-5p in the A profile), speculating that this miRs may play a role in overexpression of profibrotic gene ADAM12 in SSc-MSCs and driving these cells to a profibrotic fate.

In conclusion, the comparison of miR profiles of MSCs isolated from BM and A-tissue obtained from SSc patients, identified 2 different signatures and sharing similar functional alterations. The *in silico* prediction showed that both the BM- and A-MSCs display a similar senescent and profibrotic signature. Interestingly, both the MSCs source showed increased activity of the pathways related to survival ability and activation of compensatory mechanism, and these pathways may help MSCs to survive under the pathological constraints specific of the disease, without reverse the profibrotic phenotype, but this in silico approach is not able to define a hierarchy among these pathways and further studies need to define this setting. It must be pointed out that among the validated genes, potentially up-regulated in these cells, there are some genes involved in the angiogenic mechanisms. The activation of these genes may be involved in the improvement of the vascular conditions observed in the clinical trials, after infusion of these cells in SSc patients. In addition, “TGF-beta Signalling Pathway” is potentially up-regulated in both BM- and A-SSc-MSCs, correlating with previous work suggesting an activation of this pathway during SSc^20,30^. It has been showed that the increase of TGF-beta in SSc-MSCs may stimulate fibrotic process and also promote Tregs induction^20^, however future studies are ongoing in order to confirm the involvement of these down-regulated miRs in controlling TGF-beta function. In fact, the limitation of any *in silico* study is that the gene target interaction is a bioinformatics prediction, but it is the first necessary step, to select the potential genes, before starting focused experiments, thus restricting the field of interest and saving from unnecessary experiments. Further studies are ongoing in our laboratory to evaluate *in vitro* the ability of dysregulated miRs, to modulate the expression of the gene target and to evaluate the biological impact of this miRs on therapeutic function of SSc-MSCs. However, the main benefit of this *in silico* approach is the possibility to analyse all the identified miRs as a group, more than considering these molecules, separately, providing a larger vision of the biological pathways induced by the whole signature and give an integrated representation of the possible functional differences between BM- and A-MSCs. This bioinformatics approaches substantiate the concept of disease inherent abnormalities of SSc-MSCs, suggesting that these cells might contribute to the disease progression, as already reported in previous papers^3,22,23^. From a translational point of view, a better knowledge of MSCs signature, might allow us, in the future, to better select and potentially manipulate the MSCs to improve the development of MSC-based therapy for SSc.

## Methods

All methods were performed in accordance with the relevant guidelines and regulations.

### Patients, controls

We enrolled in this study 6 SSc patients, with a very similar clinical pattern. All patients fulfilled the 2013 classification criteria for SSc^58^. Our patients fulfilled the classification criteria in less than one year from the onset of Raynaud’s Phenomenon (RP). All SSc patients underwent 20-day washout from any immunosuppressive treatment and one month from intravenous prostanoids. During this period, only proton-pump inhibitors and clebopride were allowed. Patients who could not undergo therapeutic washout, due to severe organ complications, were not enrolled in the study. The study was approved by our local ethics committee (ASL Avezzano Sulmona L’Aquila, protocol number 1092). Demographic and clinical characteristics of the patients are showed in Table 1.

### Isolation, culture and immunophenotyping of BM-MSCs

After approval of local ethics committee (ASL Avezzano Sulmona L’Aquila n.1092) and written informed consent from patients, the BM was obtained by aspiration from the posterior superior iliac crest from 3 out of the 6 patients enrolled in the study. Three frozen BM-MSCs samples obtained from age- and sex- matched healthy donors (HC) were purchased from Lonza (USA) and used as control. Samples were placed into tubes containing ethylenediamine tetra acetic acid (EDTA) and the BM cells were obtained by density gradient sedimentation on 12% hydroxyethyl amide. The upper phase was harvested, centrifuged at 700 g for 10 min and plated at a concentration of 5 × 10^3^ cells/cm^2^ in Dulbecco’s modified Eagle’s medium (DMEM; Gibco, USA) supplemented with 10% fetal bovine serum (FBS; Gibco, USA), 2 mmol/l L-glutamine (EuroClone, Italy) and 100 U penicillin, 1000 U streptomycin (Biochrom AG, Germany). Both SSc and healthy control (HC) cultures were grown and expanded in flasks at 37 °C, 5% CO2 in a humidified thermostat for 2 weeks until confluence and used for the following experiments at third passage (P3). The International Society for Stem Cell Therapy (ISCT) drew the surface antigen pattern for defining MSCs^59^. According, the P3 isolated cells were analyzed for the surface antigen by flow cytometry (FACSCAN, Becton Dickinson, USA), to assess their purity (CD105, CD73 and CD90 > 95% positive and CD34-, CD45-, CD14-, CD79-, HLA class II (DR) ≤ 2% positive). This assessment was done concurrently with miRs evaluation.

### Isolation, culture and immunophenotyping of A-MSCs

Adipose tissue was obtained from the other 3 patients enrolled in the study, after approval of local ethics committee (ASL Avezzano Sulmona L’Aquila n.1092) and written informed consent from patients. Three frozen A-MSCs samples obtained from age- and sex- matched healthy donors (HC) were purchased from Lonza (USA) and used as control. Lipoaspirate (500 mg) was washed to remove excess blood by mixing with an equal volume of phosphate-buffered saline (PBS; Euroclone, Italy). The fat is placed in a sterile tissue culture plate with 0.075% Collagenase Type I prepared in PBS containing 2% P/S for tissue digestion (lysis buffer). Furthermore, the pellet, containing the A-MSCs, was obtained by centrifuging the sample at 2000 rpm for 5 min. After spinning, all the collagenase solution was aspirated, and the pellet resuspended in 1 ml lysis buffer, incubated for 10 min on ice, washed with 20 ml of lysis buffer and centrifuged at 2000 rpm for 5 min. The cell pellet was resuspended in a maximum of 3 ml of stromal medium (DMEM + 20%FBS + glutamine + penicillin/streptomycin), the cell suspension is filtered through 70 μm cell strainer and incubate at 37 °C with 5% humidified CO_2_. The outgrown cells were cultured in fresh medium for another 7–10 days until a confluent monolayer was obtained.

These initial cells, referred to as passage 1 (P1), were further sub-cultured at a seeding density of 100–500 cells/cm^2^ and serially passaged in a humidified thermostat for 2 weeks until confluence and used for the following experiments at third passage (P3). The P3 isolated cells were analyzed for the surface antigen by flow cytometry (FACSCAN, Becton Dickinson, USA), to assess their purity (CD105, CD73 and CD90 > 95% positive and CD34-, CD45-, CD14-, CD79-, HLA class II (DR) ≤ 2% positive). This assessment was done concurrently with miRs evaluation.

### MiRs profiling

Total RNAs were extracted from P3-MSCs using the mirVana miRs isolation kit (Ambion, Thermo Fisher Scientific, USA) by following the manufacturer’s instructions. RNA concentrations and qualities were evaluated by using NanoDrop 2000 (Thermo scientific, USA). Total RNA samples, containing the fraction less than 200 nucleotides, were used for miRs profiling studies. Identical amounts of RNAs extracted from each patient and healthy control were pooled together and subjected (700 ng per RNAs’ pool) to qRT-PCR by using the TaqMan MicroRNA reverse transcription kit (Applied Biosystems, USA) and the Megaplex RT primers human pool (Applied Biosystems, USA). Subsequently, microfluidic cards TaqMan array human microRNA A + B v3.0 (Applied Biosystems, USA) were used, according to the manufacturer’s instructions. Three replicates for each pooled sample were analysed. MiRs’ expression levels were evaluated by comparative assay: samples were analysed on a ViiA7 (Applied Biosystems, USA) and data were processed by ViiA7 software and further elaborated by Expression Suite (v.1.0.3, Applied Biosystems, USA) also at the statistical level. 2^−ΔΔCt^ method was used to determine the relative miRs’ expression levels. U6 snRNA was used as endogenous control. Significant miRs expression changes were identified using a threshold of p < 0.05 (p value calculation based on 2^−dCt^).

### Pathway analysis and target gene prediction

To gain insight into the biological function of these miRs, we performed bibliographic research and a more global approach based on *in silico* analysis. To achieve this, we used the DIANA miRPath v.3 software, which is a web-based computational tool, provided by http://diana.cslab.ece.ntua.gr/pathways/ ^60^ and designed to predict the miRs target genes.

## Supplementary information

Supplementary S1

Supplementary Table S2

## Data Availability

The datasets generated during and/or analysed during the current study are available from the corresponding author on reasonable request.

## References

[1] Altorok N, Wang Y, Kahaleh B (2014). Endothelial dysfunction in systemic sclerosis. Curr Opin Rheumatol..

[2] Cipriani P (2015). The Endothelial-mesenchymal Transition in Systemic Sclerosis Is Induced by Endothelin-1 and Transforming Growth Factor-β and May Be Blocked by Macitentan, a Dual Endothelin-1 Receptor Antagonist. J Rheumatol..

[3] Cipriani P (2014). Impaired endothelium-mesenchymal stem cells cross-talk in systemic sclerosis: a link between vascular and fibrotic features. Arthritis Res Ther..

[4] Di Benedetto P (2018). Blocking CD248 molecules in perivascular stromal cells of patients with systemic sclerosis strongly inhibits their differentiation toward myofibroblasts and proliferation: a new potential target for antifibrotic therapy. Arthritis Res Ther..

[5] Cipriani P (2015). Macitentan inhibits the transforming growth factor-β profibrotic action, blocking the signaling mediated by the ETR/TβRI complex in systemic sclerosis dermal fibroblasts. Arthritis Res Ther..

[6] Cipriani P (2013). Scleroderma Mesenchymal Stem Cells display a different phenotype from healthy controls; implications for regenerative medicine. Angiogenesis..

[7] Cipriani P (2011). Cellular players in angiogenesis during the course of systemic sclerosis. Autoimmun Rev..

[8] Del Papa N (2006). Bone marrow endothelial progenitors are defective in systemic sclerosis. Arthritis Rheum..

[9] Giacomelli R (2017). Interstitial lung disease in systemic sclerosis: current and future treatment. Rheumatol Int..

[10] Gyger G, Baron M (2015). Systemic Sclerosis: Gastrointestinal Disease and Its Management. Rheum Dis Clin North Am..

[11] Woodworth TG, Suliman YA, Li W, Furst DE, Clements P (2018). Scleroderma renal crisis and renal involvement in systemic sclerosis. Nat Rev Nephrol..

[12] Di Cesare E (2013). Early assessment of sub-clinical cardiac involvement in systemic sclerosis (SSc) using delayed enhancement cardiac magnetic resonance (CE-MRI). Eur J Radiol..

[13] Del Papa N (2018). Autologous Hematopoietic Stem Cell Transplantation for Treatment of Systemic Sclerosis. Front Immunol..

[14] Capelli C (2017). Phenotypical and Functional Characteristics of *In Vitro*-Expanded Adipose-Derived Mesenchymal Stromal Cells From Patients With Systematic Sclerosis. Cell Transplant..

[15] Hosseinikia R (2017). Molecular and Cellular Interactions of Allogenic and Autologus Mesenchymal Stem Cells with Innate and Acquired Immunity and Their Role in Regenerative Medicine. Int J Hematol Oncol Stem Cell Res.

[16] Cipriani P (2016). Perivascular Cells in Diffuse Cutaneous Systemic Sclerosis Overexpress Activated ADAM12 and Are Involved in Myofibroblast Transdifferentiation and Development of Fibrosis. J Rheumatol..

[17] Cipriani P (2015). Mesenchymal stromal cells and rheumatic diseases: new tools from pathogenesis to regenerative therapies. Cytotherapy..

[18] Cipriani P, Ruscitti P, Giacomelli R (2015). Stem cell therapies for systemic sclerosis. Br J Haematol..

[19] Moroncini G (2018). Mesenchymal stromal cells from human umbilical cord prevent the development of lung fibrosis in immunocompetent mice. PLoS One..

[20] Cipriani P (2013). Mesenchymal stem cells (MSCs) from scleroderma patients (SSc) preserve their immunomodulatory properties although senescent and normally induce T regulatory cells (Tregs) with a functional phenotype: implications for cellular-based therapy. Clin Exp Immunol..

[21] Griffin M (2017). Characteristics of human adipose derived stem cells in scleroderma in comparison to sex and age matched normal controls: implications for regenerative medicine. Stem Cell Res Ther..

[22] Maria AT (2017). Adipose-Derived Mesenchymal Stem Cells in Autoimmune Disorders: State of the Art and Perspectives for Systemic Sclerosis. Clin Rev Allergy Immunol..

[23] Hegner B (2016). Intrinsic Deregulation of Vascular Smooth Muscle and Myofibroblast Differentiation in Mesenchymal Stromal Cells from Patients with Systemic Sclerosis. PLoS One..

[24] Barbado J, Tabera S, Sánchez A, García-Sancho J (2018). Therapeutic potential of allogeneic mesenchymal stromal cells transplantation for lupus nephritis. Lupus..

[25] Liang J (2018). Safety analysis in patients with autoimmune disease receiving allogeneic mesenchymal stem cells infusion: a long-term retrospective study. Stem Cell Res Ther..

[26] Reid G, Kirschner MB, van Zandwijk N (2011). Circulating microRNAs: Association with disease and potential use as biomarkers. Crit Rev Oncol Hematol..

[27] Bartel DP (2004). miRNAs: genomics, biogenesis, mechanism, and function. Cell.

[28] Gong F (2018). Neo4j graph database realizes efficient storage performance of oilfield ontology. PLoS One..

[29] Giacomelli, R. *et al*. International consensus: What else can we do to improve diagnosis and therapeutic strategies in patients affected by autoimmune rheumatic diseases (rheumatoid arthritis, spondyloarthritides, systemic sclerosis, systemic lupus erythematosus, antiphospholipid syndrome and Sjogren’s syndrome)?: The unmet needs and the clinical grey zone in autoimmune disease management. *Autoimmun Rev*. **16**, 911–924 (2017). 10.1016/j.autrev.2017.07.012 28705780

[30] Sacchetti C (2017). PTP4A1 promotes TGFβ signaling and fibrosis in systemic sclerosis. Nat Commun..

[31] Raman M, Earnest S, Zhang K, Zhao Y, Cobb MH (2007). TAO kinases mediate activation of p38 in response to DNA damage. EMBO J..

[32] Chen P (2014). BRCA1 silencing is associated with failure of DNA repairing in retinal neurocytes. PLoS One..

[33] Bigot N (2015). Hypoxia Differentially Modulates the Genomic Stability of Clinical-Grade ADSCs and BM-MSCs in Long-Term Culture. Stem Cells..

[34] Del Papa N (2015). Autologous fat grafting in the treatment of fibrotic perioral changes in patients with systemic sclerosis. Cell Transplant..

[35] Peltzer J (2018). Mesenchymal Stromal Cells Based Therapy in Systemic Sclerosis: Rational and Challenges. Front. Immunol..

[36] Mun CH, Kang MI, Shin YD, Kim Y, Park YB (2018). The Expression of Immunomodulation-Related Cytokines and Genes of Adipose- and Bone Marrow-Derived Human Mesenchymal Stromal Cells from Early to Late Passages. Tissue Eng. Regen. Med..

[37] Zheng Y (2018). Comparative analysis of MicroRNA expression in dog lungs infected with the H3N2 and H5N1 canine influenza viruses. Microb Pathog..

[38] Kanehisa M, Sato Y, Kawashima M, Furumichi M, Tanabe M (2016). KEGG as a reference resource for gene and protein annotation. Nucleic Acids Res..

[39] Xu Q (2016). Overexpression of KLF4 promotes cell senescence through microRNA-203-survivin-p21 pathway. Oncotarget..

[40] Li T (2018). Human Umbilical Cord Mesenchymal Stem Cells Protect Against SCA3 by Modulating the Level of 70 kD Heat Shock Protein. Cell Mol Neurobiol..

[41] Magnuson B, Ekim B, Fingar DC (2012). Regulation and function of ribosomal protein S6 kinase (S6K) within mTOR signalling networks. Biochem J..

[42] Zeng Y (2015). Pak2 regulates hematopoietic progenitor cell proliferation, survival, and differentiation. Stem Cells..

[43] Jiang D (2017). Phospholipase Cγ1 Mediates Intima Formation Through Akt-Notch1 Signaling Independent of the Phospholipase Activity. J Am Heart Assoc..

[44] Yi T, Papadopoulos E, Hagner PR, Wagner G (2013). Hypoxia-inducible factor-1α (HIF-1α) promotes cap-dependent translation of selective mRNAs through up-regulating initiation factor eIF4E1 in breast cancer cells under hypoxia conditions. Biol Chem..

[45] Heikkinen PT (2010). Hypoxia-activated Smad3-specific dephosphorylation by PP2A. J Biol Chem..

[46] Sonntag R (2018). Cyclin E1 and cyclin-dependent kinase 2 are critical for initiation, but not for progression of hepatocellular carcinoma. Proc Natl Acad Sci USA.

[47] Sun HJ (2006). A proteomic analysis during serial subculture and osteogenic differentiation of human mesenchymal stem cell. J Orthop Res..

[48] Chen H (2012). Anti-fibrotic effects via regulation of transcription factor Sp1 on hepatic stellate cells. Cell Physiol Biochem.

[49] Zhao XR, Zhang MC, Xie HT, Ji N, Sun LT (2018). p70S6K activation promotes the transdifferentiation of fibroblasts to myofibroblasts in pterygium tissue growth on the cornea. Biotechnol Lett..

[50] Bang OS, Ruscetti FW, Lee MH, Kim SJ, Birchenall-Roberts MC (1996). Transforming growth factor-beta1 modulates p107 function in myeloid cells: correlation with cell cycle progression. J Biol Chem..

[51] Cervantes S (2017). Late-stage differentiation of embryonic pancreatic β-cells requires Jarid2. Sci Rep..

[52] Lv D (2017). Histone Acetyltransferase KAT6A Upregulates PI3K/AKT Signaling through TRIM24 Binding. Cancer Res..

[53] Seira O, Del Río JA (2014). Glycogen synthase kinase 3 beta (GSK3β) at the tip of neuronal development and regeneration. Mol Neurobiol..

[54] Mahmoud AI (2013). Meis1 regulates postnatal cardiomyocyte cell cycle arrest. Nature..

[55] Doi R (2014). Critical role of Frizzled1 in age-related alterations of Wnt/β-catenin signal in myogenic cells during differentiation. Genes Cells..

[56] Zhong X (2016). Suppression of MicroRNA 200 Family Expression by Oncogenic KRAS Activation Promotes Cell Survival and Epithelial-Mesenchymal Transition in KRAS-Driven Cancer. Mol Cell Biol..

[57] Dulauroy S, Di Carlo SE, Langa F, Eberl G, Peduto L (2012). Lineage tracing and genetic ablation of ADAM12+ perivascular cells identify a major source of profibrotic cells during acute tissue injury. Nat. Med..

[58] van den Hoogen F (2013). Classification criteria for systemic sclerosis: an American college of rheumatology/European league against rheumatism collaborative initiative. Ann Rheum Dis.

[59] Dominici M (2006). Minimal criteria for defining multipotent mesenchymal stromal cells. The International Society for Cellular Therapy position statement. Cytotherapy..

[60] Vlachos IS (2015). DIANA-miRPath v3.0: deciphering microRNA function with experimental support. Nucleic Acids Res..

