# Supplementary material for: Mesenchymal stem cells of Systemic Sclerosis patients, derived from different sources, show a profibrotic microRNA profiling
Source: Sci Rep. 2019 May 9;9:7144. doi: 10.1038/s41598-019-43638-0 (PMC6509164; doi:10.1038/s41598-019-43638-0)
Supplement: Supplementary file 2 — Supplementary Table S2 [file 41598_2019_43638_MOESM2_ESM.doc]

**Title:** Mesenchymal stem cells of Systemic Sclerosis patients, derived from different sources, show a profibrotic microRNA profiling.

**Authors:** Paola Di Benedetto, Noemi Panzera, Paola Cipriani, Valentina Mastroiaco, Alessandra Tessitore, Vasiliki Liakouli, Piero Ruscitti, Onorina Berardicurti, Francesco Carubbi, Giuliana Guggino, Andrea Bianchi, Antinisca Di Marco, Francesco Ciccia, Edoardo Alesse, Roberto Giacomelli

**Supplementary Table S2. DIANA MicroT-CDS Predicted Interactions**

The genes target interactions of significantly down- or up-regulated miRs, reported in DIANA MicroT-CDS. The score was calculated by DIANA miRPath v.3 software.

| **Profile BM (miRs down-regulated)** |  |  |  |
| --- | --- | --- | --- |
| **Signalling Pathways Regulating Pluripotency Of Stem Cells (Hsa04550)** |  |  |  |
| hsa-miR-519b-3p | Gene Name | Score | Experimentally Validated |
|  | STAT3 | 0.873 | No |
|  | REST | 0.839 | No |
|  | FZD6 | 0.844 | No |
|  | INHBA | 0.906 | No |
|  | ZFHX3 | 0.890 | No |
|  | FZD3 | 0.846 | No |
|  | RIF1 | 0.912 | No |
|  | SMAD5 | 0.851 | No |
|  | ACVR1C | 0.807 | No |
|  | IGF1 | 0.847 | No |
|  | NEUROG1 | 0.889 | No |
|  | MAPK1 | 0.828 | No |
|  | JAK1 | 0.976 | No |
|  | BMPR2 | 0.944 | No |
|  | COMMD3-BMI1 | 0.850 | No |
| hsa-miR-483-3p | Gene Name | Score | Experimentally validated |
|  | MAPK11 | 0.865 | No |
|  | IGF1 | 0.997 | No |
|  | BMPR1A | 0.843 | No |
|  | HNF1A | 0.802 | No |
|  | PIK3R2 | 0.869 | No |
| hsa-miR-200b-3p | Gene Name | Score | Experimentally validated |
|  | WNT16 | 0.949 | No |
|  | PAX6 | 0.941 | No |
|  | SMAD2 | 0.864 | Yes |
|  | SMARCAD1 | 0.848 | No |
|  | INHBA | 0.928 | No |
|  | RIF1 | 0.845 | No |
|  | LIFR | 0.856 | No |
|  | SMAD5 | 0.846 | Yes |
|  | ACVR1C | 0.882 | No |
|  | AKT3 | 0.866 | No |
|  | PIK3CA | 0.930 | No |
|  | KLF4 | 0.873 | Yes |
| hsa-miR-489-3p | Gene Name | Score | Experimentally validated |
|  | MAP2K1 | 0.825 | No |
| hsa-miR-875-5p | Gene Name | Score | Experimentally validated |
|  | ZFHX3 | 0.992 | No |
| **MAPK Signalling Pathway (Hsa04010)** |  |  |  |
| hsa-miR-200b-3p | Gene Name | Score | Experimentally validated |
|  | NTF3 | 0.890 | No |
|  | CRKL | 0.993 | Yes |
|  | STK4 | 0.878 | No |
|  | RASA2 | 0.990 | Yes |
|  | ELK4 | 0.932 | Yes |
|  | PAK2 | 0.840 | Yes |
|  | MAP2K5 | 0.857 | No |
|  | MAP4K3 | 0.996 | No |
|  | MAP4K4 | 0.970 | No |
|  | EGFR | 0.914 | No |
|  | MAP3K1 | 0.977 | No |
|  | BDNF | 0.966 | No |
|  | RRAS2 | 0.850 | No |
|  | TAOK1 | 0.902 | Yes |
|  | IKBKB | 0.893 | No |
|  | JUN | 0.988 | Yes |
|  | RAPGEF2 | 0.970 | No |
|  | PPM1A | 0.880 | No |
|  | SOS1 | 0.966 | No |
|  | RPS6KA3 | 0.921 | Yes |
|  | AKT3 | 0.866 | No |
|  | MAP3K2 | 0.848 | No |
|  | CACNB2 | 0.968 | No |
|  | SRF | 0.911 | No |
|  | RAP1B | 0.996 | Yes |
|  | DUSP1 | 0.998 | Yes |
| hsa-miR-483-3p | Gene Name | Score | Experimentally validated |
|  | MAP2K6 | 0.903 | No |
|  | MAPK8 | 0.807 | No |
|  | ZAK | 0.808 | Yes |
|  | PDGFB | 0.953 | No |
|  | MAPK11 | 0.865 | No |
| hsa-miR-519b-3p | Gene Name | Score | Experimentally validated |
|  | TAOK3 | 0.990 | No |
|  | PDGFRA | 0.935 | No |
|  | ELK4 | 0.832 | No |
|  | RASGRF2 | 0.805 | No |
|  | PPP3R1 | 0.839 | No |
|  | MAP3K1 | 0.852 | No |
|  | TAOK1 | 0.853 | No |
|  | PPP3CA | 0.909 | No |
|  | RAPGEF2 | 0.808 | No |
|  | MAPK8 | 0.862 | No |
|  | SOS1 | 0.960 | No |
|  | FGF9 | 0.899 | No |
|  | STK3 | 0.908 | No |
|  | HSPA8 | 0.950 | Yes |
|  | RPS6KA3 | 0.906 | No |
|  | MAP3K2 | 0.985 | No |
|  | MAPK1 | 0.828 | No |
|  | TGFBR2 | 0.997 | No |
|  | MAP3K5 | 0.974 | No |
| hsa-miR-875-5p | Gene Name | Score | Experimentally validated |
|  | FGF10 | 0.801 | No |
|  | EGFR | 0.925 | No |
|  | TAOK1 | 0.832 | No |
|  | MEF2C | 0.926 | No |
|  | PRKACB | 0.854 | No |
|  | TGFB3 | 0.997 | No |
| hsa-miR-489-3p | Gene Name | Score | Experimentally validated |
|  | MAP2K1 | 0.825 | No |
| **HIF-1 Signalling Pathway (Hsa04066)** |  |  |  |
| hsa-miR-519b-3p | Gene Name | Score | Experimentally validated |
|  | STAT3 | 0.873 | No |
|  | HIF1A | 0.936 | No |
|  | IGF1 | 0.847 | No |
|  | EIF4E2 | 0.954 | No |
|  | CDKN1A | 0.895 | No |
|  | MAPK1 | 0.828 | No |
| hsa-miR-200b-3p | Gene Name | Score | Experimentally validated |
|  | CUL2 | 0.813 | No |
|  | EGFR | 0.914 | No |
|  | TLR4 | 0.819 | No |
|  | PLCG1 | 0.930 | Yes |
|  | FLT1 | 0.985 | Yes |
|  | PDK1 | 0.859 | No |
|  | EP300 | 0.825 | No |
|  | AKT3 | 0.866 | No |
|  | EIF4E2 | 0.970 | Yes |
|  | PIK3CA | 0.930 | No |
|  | RPS6KB1 | 1.000 | No |
|  | EGLN1 | 0.983 | No |
| hsa-miR-875-5p | Gene Name | Score | Experimentally validated |
|  | EGFR | 0.925 | No |
|  | HKDC1 | 0.811 | No |
| hsa-miR-483-3p | Gene Name | Score | Experimentally validated |
|  | INSR | 0.828 | No |
|  | PLCG2 | 0.900 | No |
|  | IGF1 | 0.997 | No |
|  | PIK3R2 | 0.869 | No |
| hsa-miR-489-3p | Gene Name | Score | Experimentally validated |
|  | MAP2K1 | 0.825 | No |
| hsa-miR-642a-5p | Gene Name | Score | Experimentally validated |
|  | HMOX1 | 0.872 | No |
| **TGF-beta Signalling Pathway (Hsa04350)** |  |  |  |
| hsa-miR-875-5p | Gene Name | Score | Experimentally validated |
|  | TGFB3 | 0.997 | No |
| hsa-miR-519b-3p | Gene Name | Score | Experimentally validated |
|  | INHBA | 0.906 | No |
|  | ZFYVE9 | 0.947 | No |
|  | SMAD5 | 0.851 | No |
|  | ACVR1C | 0.807 | No |
|  | SMAD7 | 0.927 | No |
|  | MAPK1 | 0.828 | No |
|  | TGFBR2 | 0.997 | No |
|  | BMPR2 | 0.944 | No |
| hsa-miR-200b-3p | Gene Name | Score | Experimentally validated |
|  | SMAD2 | 0.864 | Yes |
|  | INHBA | 0.928 | No |
|  | SMAD5 | 0.846 | Yes |
|  | ACVR1C | 0.882 | No |
|  | EP300 | 0.825 | No |
|  | NOG | 0.997 | No |
|  | PPP2R1B | 0.864 | Yes |
|  | RPS6KB1 | 1.000 | No |
| hsa-miR-483-3p | Gene Name | Score | Experimentally validated |
|  | BMPR1A | 0.843 | No |
| **P53 Signalling Pathway (Hsa04115)** |  |  |  |
| hsa-miR-200b-3p | Gene Name | Score | Experimentally validated |
|  | ZMAT3 | 0.853 | Yes |
|  | CDK2 | 0.865 | Yes |
|  | APAF1 | 0.876 | No |
|  | PMAIP1 | 0.989 | Yes |
|  | CCNE2 | 0.855 | Yes |
|  | TP73 | 0.885 | No |
|  | SESN1 | 1.000 | Yes |
|  | SIAH1 | 0.806 | Yes |
|  | SESN3 | 0.991 | No |
| hsa-miR-519b-3p | Gene Name | Score | Experimentally validated |
|  | CDK2 | 0.919 | No |
|  | RCHY1 | 0.818 | No |
|  | MDM4 | 0.881 | No |
|  | IGF1 | 0.847 | No |
|  | CDKN1A | 0.895 | No |
|  | SESN3 | 0.891 | No |
|  | PTEN | 0.956 | Yes |
|  | CCNG2 | 0.808 | No |
| hsa-miR-483-3p | Gene Name | Score | Experimentally validated |
|  | EI24 | 0.959 | No |
|  | IGF1 | 0.997 | No |
| hsa-miR-875-5p | Gene Name | Score | Experimentally validated |
|  | MDM4 | 0.993 | No |
| **PI3K-Akt Signalling Pathway (Hsa04151)** |  |  |  |
| hsa-miR-642a-5p | Gene Name | Score | Experimentally validated |
|  | CREB1 | 0.801 | No |
|  | COL5A3 | 0.845 | No |
| hsa-miR-519b-3p | Gene Name | Score | Experimentally validated |
|  | PHLPP2 | 0.924 | No |
|  | PDGFRA | 0.935 | No |
|  | MYB | 0.838 | No |
|  | ITGB8 | 0.894 | No |
|  | CREB5 | 0.856 | No |
|  | CDK2 | 0.919 | No |
|  | ITGB4 | 0.902 | No |
|  | PIK3AP1 | 0.889 | No |
|  | CREB1 | 0.859 | No |
|  | PPP2R2A | 0.946 | No |
|  | SOS1 | 0.960 | No |
|  | FGF9 | 0.899 | No |
|  | CHRM2 | 0.977 | No |
|  | IGF1 | 0.847 | No |
|  | EIF4E2 | 0.954 | No |
|  | PDGFD | 0.968 | No |
|  | OSM | 0.897 | No |
|  | CDKN1A | 0.895 | No |
|  | PTEN | 0.956 | Yes |
|  | MAPK1 | 0.828 | No |
|  | CSF1 | 0.838 | No |
|  | JAK1 | 0.976 | No |
| hsa-miR-200b-3p | Gene Name | Score | Experimentally validated |
|  | PPP2R5E | 0.971 | Yes |
|  | PPP2R2C | 0.897 | No |
|  | YWHAG | 0.985 | Yes |
|  | CDK2 | 0.865 | Yes |
|  | YWHAB | 0.811 | No |
|  | MTCP1 | 0.886 | No |
|  | EGFR | 0.914 | No |
|  | TLR4 | 0.819 | No |
|  | YWHAQ | 0.926 | No |
|  | IKBKB | 0.893 | No |
|  | PTK2 | 0.910 | Yes |
|  | G6PC | 0.927 | No |
|  | BRCA1 | 0.875 | Yes |
|  | ITGAV | 0.804 | Yes |
|  | CCNE2 | 0.855 | Yes |
|  | RELN | 0.988 | No |
|  | FLT1 | 0.985 | Yes |
|  | COL4A3 | 0.855 | No |
|  | SOS1 | 0.966 | No |
|  | CHRM2 | 0.850 | No |
|  | AKT3 | 0.866 | No |
|  | EIF4E2 | 0.970 | Yes |
|  | PIK3CA | 0.930 | No |
|  | FN1 | 0.867 | No |
|  | PPP2R1B | 0.864 | Yes |
|  | KDR | 0.998 | Yes |
|  | RPS6KB1 | 1.000 | No |
|  | COL4A1 | 0.827 | No |
|  | EFNA1 | 0.996 | Yes |
| hsa-miR-483-3p | Gene Name | Score | Experimentally validated |
|  | PKN3 | 0.824 | No |
|  | PDGFB | 0.953 | No |
|  | INSR | 0.828 | No |
|  | IGF1 | 0.997 | No |
|  | PIK3R2 | 0.869 | No |
| hsa-miR-875-5p | Gene Name | Score | Experimentally validated |
|  | CREB5 | 0.812 | No |
|  | FGF10 | 0.801 | No |
|  | EGFR | 0.925 | No |
|  | IRS1 | 0.882 | No |
| hsa-miR-489-3p | Gene Name | Score | Experimentally validated |
|  | MAP2K1 | 0.825 | No |
| **Profile BM (miRs up-regulated)** |  |  |  |
| **ECM-Receptor Interaction (Hsa04512)** |  |  |  |
| hsa-miR-629-3p | Gene Name | Score | Experimentally validated |
|  | SV2B | 0.824 | No |
|  | COL24A1 | 0.983 | No |
|  | COL27A1 | 0.950 | No |
|  | COL6A6 | 0.955 | No |
|  | COL4A4 | 0.938 | No |
|  | FN1 | 0.931 | No |
|  | COL4A1 | 0.820 | No |
| hsa-miR-432-5p | Gene Name | Score | Experimentally validated |
|  | COL4A5 | 1.000 | No |
|  | COL5A2 | 0.866 | No |
|  | CD47 | 0.901 | No |
| **Profile A (miRs down-regulated)** |  |  |  |
| **TGF-beta Signalling Pathway (Hsa04350)** |  |  |  |
| hsa-miR-155-5p | Gene Name | Score | Experimentally validated |
|  | SMAD2 | 0.924 | No |
|  | PPP2CA | 0.811 | No |
|  | GDF6 | 0.924 | No |
|  | SP1 | 0.923 | Yes |
|  | ACVR1C | 0.826 | No |
|  | RPS6KB1 | 0.858 | No |
| hsa-miR-135b-5p | Gene Name | Score | Experimentally validated |
|  | ROCK1 | 0.991 | No |
|  | SMAD2 | 0.891 | No |
|  | NODAL | 0.805 | No |
|  | ACVR1B | 0.890 | No |
|  | INHBA | 0.867 | No |
|  | SMAD5 | 0.993 | No |
|  | SP1 | 0.896 | No |
|  | RBX1 | 0.821 | No |
| hsa-miR-708-5p | Gene Name | Score | Experimentally validated |
|  | THBS1 | 0.921 | No |
|  | SMAD3 | 0.829 | No |
| hsa-miR-500a-5p | Gene Name | Score | Experimentally validated |
|  | TFDP1 | 0.878 | No |
|  | TGFB2 | 0.807 | No |
|  | BMPR1A | 0.882 | No |
|  | BAMBI | 0.986 | No |
|  | RPS6KB1 | 0.916 | Yes |
| hsa-miR-146a-5p | Gene Name | Score | Experimentally validated |
|  | SMAD4 | 0.888 | Yes |
|  | RBL1 | 0.948 | Yes |
| hsa-miR-146b-5p | Gene Name | Score | Experimentally validated |
|  | SMAD4 | 0.900 | No |
|  | RBL1 | 0.898 | Yes |
| hsa-miR-199b-5p | Gene Name | Score | Experimentally validated |
|  | ACVR2B | 0.894 | No |
|  | RBL1 | 0.822 | No |
|  | SP1 | 0.854 | No |
|  | TGFB2 | 0.994 | No |
| hsa-miR-204-5p | Gene Name | Score | Experimentally validated |
|  | BMPR1A | 0.834 | No |
| **Signalling Pathways Regulating Pluripotency Of Stem Cells (Hsa04550)** |  |  |  |
| hsa-miR-199b-5p | Gene Name | Score | Experimentally validated |
|  | GSK3B | 0.892 | No |
|  | FZD6 | 0.833 | No |
|  | ACVR2B | 0.894 | No |
|  | FZD4 | 0.959 | No |
| hsa-miR-204-5p | Gene Name | Score | Experimentally validated |
|  | FZD1 | 0.823 | Yes |
|  | BMPR1A | 0.834 | No |
|  | MEIS1 | 0.869 | Yes |
| hsa-miR-155-5p | Gene Name | Score | Experimentally validated |
|  | JARID2 | 1.000 | Yes |
|  | GSK3B | 0.843 | Yes |
|  | FZD5 | 0.806 | No |
|  | KAT6A | 0.830 | Yes |
|  | SMAD2 | 0.924 | No |
|  | KRAS | 0.974 | Yes |
|  | POU5F1B | 0.823 | No |
|  | ZIC3 | 0.994 | No |
|  | ACVR1C | 0.826 | No |
|  | PIK3CA | 0.818 | No |
|  | MEIS1 | 0.995 | No |
| hsa-miR-135b-5p | Gene Name | Score | Experimentally validated |
|  | GSK3B | 0.896 | No |
|  | KAT6A | 0.816 | No |
|  | SMAD2 | 0.891 | No |
|  | NODAL | 0.805 | No |
|  | ACVR1B | 0.890 | No |
|  | PIK3R2 | 0.944 | No |
|  | SMARCAD1 | 0.941 | Yes |
|  | INHBA | 0.867 | No |
|  | WNT3 | 0.874 | No |
|  | PIK3CD | 0.805 | No |
|  | JAK2 | 0.928 | No |
|  | SMAD5 | 0.993 | No |
|  | FZD1 | 0.840 | No |
|  | IL6ST | 0.834 | No |
|  | KLF4 | 0.886 | No |
|  | PIK3R2 | 0.925 | No |
| hsa-miR-500a-5p | Gene Name | Score | Experimentally validated |
|  | FZD3 | 0.817 | No |
|  | BMPR1A | 0.882 | No |
|  | COMMD3-BMI1 | 0.985 | No |
| hsa-miR-146a-5p | Gene Name | Score | Experimentally validated d |
|  | NRAS | 0.923 | No |
|  | APC | 0.843 | No |
|  | FZD3 | 0.883 | No |
|  | SMAD4 | 0.888 | Yes |
| hsa-miR-146b-5p | Gene Name | Score | Experimentally validated |
|  | NRAS | 0.927 | No |
|  | APC | 0.846 | No |
|  | FZD3 | 0.879 | No |
|  | SMAD4 | 0.900 | No |
| hsa-miR-708-5p | Gene Name | Score | Experimentally validated |
|  | KAT6A | 0.957 | No |
|  | APC | 0.867 | No |
|  | SMAD3 | 0.829 | No |
| hsa-miR-218-5p | Gene Name | Score | Experimentally validated |
|  | FZD6 | 0.987 | No |
|  | MYF5 | 0.896 | No |
| hsa-miR-423-5p | Gene Name | Score | Experimentally validated |
|  | PCGF3 | 0.802 | No |
| **Regulation Of Actin Cytoskeleton (Hsa04810**) |  |  |  |
| hsa-miR-146a-5p | Gene Name | Score | Experimentally validated |
|  | ARPC5 | 0.896 | No |
|  | BRAF | 0.863 | No |
|  | PDGFRA | 0.869 | No |
|  | NRAS | 0.923 | No |
|  | APC | 0.843 | No |
|  | IQGAP3 | 0.875 | No |
|  | IQGAP1 | 0.892 | No |
|  | PIP5K1B | 0.868 | No |
| hsa-miR-146b-5p | Gene Name | Score | Experimentally validated |
|  | ARPC5 | 0.884 | Yes |
|  | BRAF | 0.866 | No |
|  | PDGFRA | 0.872 | No |
|  | NRAS | 0.927 | No |
|  | APC | 0.846 | No |
|  | IQGAP3 | 0.865 | No |
|  | IQGAP1 | 0.854 | No |
|  | PIP5K1B | 0.883 | No |
| hsa-miR-155-5p | Gene Name | Score | Experimentally validated |
|  | PAK2 | 0.815 | Yes |
|  | PAK7 | 0.857 | Yes |
|  | KRAS | 0.974 | Yes |
|  | ABI2 | 0.960 | No |
|  | SOS1 | 0.935 | No |
|  | FGF9 | 0.959 | Yes |
|  | PIK3CA | 0.818 | No |
|  | VAV3 | 0.894 | No |
|  | FGF7 | 0.994 | No |
| hsa-miR-423-5p | Gene Name | Score | Experimentally validated |
|  | ARPC5 | 0.807 | Yes |
|  | CRK | 0.956 | No |
|  | ITGA5 | 0.852 | Yes |
|  | INSRR | 0.857 | No |
|  | MRAS | 0.851 | No |
|  | PPP1R12B | 0.892 | No |
|  | PAK3 | 0.934 | No |
|  | GSN | 0.861 | No |
|  | LIMK2 | 0.818 | No |
| hsa-miR-135b-5p | Gene Name | Score | Experimentally validated |
|  | ROCK1 | 0.991 | No |
|  | SSH1 | 0.865 | No |
|  | PIK3R2 | 0.944 | No |
|  | ROCK2 | 0.993 | No |
|  | PAK7 | 0.800 | No |
|  | ITGA1 | 0.837 | No |
|  | ARHGEF6 | 0.908 | No |
|  | PTK2 | 0.951 | No |
|  | ITGAV | 0.857 | No |
|  | PIK3CD | 0.805 | No |
|  | NCKAP1L | 0.839 | No |
|  | WASF2 | 0.864 | Yes |
|  | DIAPH3 | 0.831 | No |
|  | ITGA2 | 0.903 | No |
|  | PPP1R12C | 0.914 | No |
|  | ARHGEF7 | 0.944 | No |
|  | ARHGEF4 | 0.984 | No |
|  | PIP4K2C | 0.901 | No |
|  | PIK3R2 | 0.925 | No |
| hsa-miR-500a-5p | Gene Name | Score | Experimentally validated |
|  | RDX | 0.983 | No |
|  | PAK3 | 0.854 | No |
|  | PTK2 | 0.844 | No |
|  | FGF9 | 0.957 | No |
|  | CHRM2 | 0.873 | No |
|  | FGF23 | 0.875 | No |
|  | NCKAP1 | 0.916 | No |
| hsa-miR-218-5p | Gene Name | Score | Experimentally validated |
|  | WASL | 0.872 | No |
|  | PPP1CC | 0.885 | Yes |
|  | ABI2 | 0.856 | No |
|  | ACTN1 | 0.986 | Yes |
| hsa-miR-199b-5p | Gene Name | Score | Experimentally validated |
|  | SOS2 | 0.951 | No |
|  | ITGA3 | 0.865 | No |
|  | ABI2 | 0.850 | No |
|  | GIT1 | 0.929 | Yes |
| hsa-miR-204-5p | Gene Name | Score | Experimentally validated |
|  | FGF20 | 0.979 | No |
|  | ABI2 | 0.868 | No |
|  | SOS1 | 0.946 | No |
| hsa-miR-708-5p | Gene Name | Score | Experimentally validated |
|  | APC | 0.867 | No |
|  | PAK2 | 0.838 | No |
|  | IQGAP1 | 0.927 | Yes |
|  | DIAPH1 | 0.831 | Yes |
| **Wnt Signalling Pathway (Hsa04310)** |  |  |  |
| hsa-miR-135b-5p | Gene Name | Score | Experimentally validated |
|  | GSK3B | 0.896 | No |
|  | TCF7L2 | 0.965 | No |
|  | ROCK2 | 0.993 | No |
|  | WNT3 | 0.874 | No |
|  | PLCB1 | 0.850 | No |
|  | SIAH1 | 0.825 | No |
|  | FZD1 | 0.840 | No |
|  | RBX1 | 0.821 | No |
|  | MAPK10 | 0.819 | No |
| hsa-miR-199b-5p | Gene Name | Score | Experimentally validated |
|  | GSK3B | 0.892 | No |
|  | BTRC | 0.822 | No |
|  | FZD6 | 0.833 | No |
|  | NLK | 0.875 | No |
|  | FZD4 | 0.959 | No |
| hsa-miR-155-5p | Gene Name | Score | Experimentally validated |
|  | GSK3B | 0.843 | Yes |
|  | FZD5 | 0.806 | No |
|  | TCF7L2 | 0.869 | No |
|  | CHD8 | 0.950 | Yes |
|  | CSNK1A1 | 0.947 | Yes |
|  | MAPK10 | 0.836 | No |
| hsa-miR-500a-5p | Gene Name | Score | Experimentally validated |
|  | FZD3 | 0.817 | No |
|  | BAMBI | 0.986 | No |
|  | TBL1XR1 | 0.953 | Yes |
| hsa-miR-204-5p | Gene Name | Score | Experimentally validated |
|  | CAMK2D | 0.880 | No |
|  | CCND2 | 0.987 | Yes |
|  | FZD1 | 0.823 | Yes |
| hsa-miR-423-5p | Gene Name | Score | Experimentally validated |
|  | LRP5 | 0.980 | No |
|  | NFATC4 | 0.930 | No |
|  | PLCB1 | 1.000 | No |
|  | PRKACA | 0.854 | Yes |
| hsa-miR-708-5p | Gene Name | Score | Experimentally validated |
|  | BTRC | 0.856 | No |
|  | APC | 0.867 | No |
|  | SMAD3 | 0.829 | No |
|  | NFATC3 | 0.880 | No |
| hsa-miR-146a-5p | Gene Name | Score | Experimentally validated |
|  | CAMK2D | 0.846 | No |
|  | APC | 0.843 | No |
|  | FZD3 | 0.883 | No |
|  | SMAD4 | 0.888 | Yes |
|  | CXXC4 | 0.967 | No |
| hsa-miR-146b-5p | Gene Name | Score | Experimentally validated |
|  | CAMK2D | 0.851 | No |
|  | APC | 0.846 | No |
|  | FZD3 | 0.879 | No |
|  | SMAD4 | 0.900 | No |
|  | CXXC4 | 0.962 | No |
| hsa-miR-218-5p | Gene Name | Score | Experimentally validated |
|  | FZD6 | 0.987 | No |
|  | SENP2 | 0.840 | Yes |
| **Profile A (miRs up-regulated)** |  |  |  |
| **Thyroid Hormone Signalling Pathway (Hsa04919)** |  |  |  |
| hsa-miR-1225-3p | Gene Name | Score | Experimentally validated |
|  | ESR1 | 0.870 | No |
|  | THRA | 0.927 | No |
|  | MED24 | 0.921 | No |
|  | PRKACG | 0.993 | No |
|  | SLC16A2 | 0.820 | No |
|  | ITGB3 | 0.887 | No |
| hsa-miR-1227-3p | Gene Name | Score | Experimentally validated |
|  | ESR1 | 0.819 | No |
